# Response Strategies of Giant Panda, Red Panda, and Forest Musk Deer to Human Disturbance in Sichuan Liziping National Nature Reserve

**DOI:** 10.3390/biology15020194

**Published:** 2026-01-21

**Authors:** Mengyi Duan, Qinlong Dai, Wei Luo, Ying Fu, Bin Feng, Hong Zhou

**Affiliations:** 1 Liziping Giant Panda’s Ecology and Conservation Observation and Research Station of Sichuan Province, Nanchong 637009, China; 2 College of Giant Panda, China West Normal University, Nanchong 637009, China; 3 Liziping Nature Reserve Administration Bureau of Sichuan Province, Ya’an 625400, China; 4 School of Medicine, Nanjing University of Chinese Medicine, Nanjing 210023, China

**Keywords:** human disturbance, infrared camera, spatiotemporal pattern, *Ailuropoda melanoleuca*, *Ailurus fulgens*, *Moschus berezovskii*

## Abstract

The persistent expansion in the intensity and scope of human disturbance has become a key driver of global biodiversity loss, affecting wildlife behavior and population stability across multiple dimensions. As a characteristic symbiotic assemblage in the subalpine forest ecosystems of Sichuan, the giant panda (*Ailuropoda melanoleuca*), red panda (*Ailurus fulgens*), and forest musk deer (*Moschus berezovskii*) exhibit significant research value in their responses to human disturbance. However, existing studies lack systematic analysis of multiple disturbances within the same protected area. This study was conducted in the Sichuan Liziping National Nature Reserve, where infrared camera traps were deployed using a kilometer-grid layout. By integrating spatiotemporal pattern analysis and Generalized Additive Models (GAM), we investigated the characteristics of human disturbance and the response strategies of the three species within their habitats. The results show that: (1) A total of seven types of human disturbance were identified in the reserve, with the top three by frequency being cattle disturbance, goat disturbance, and walking disturbance; (2) Temporally, summer and winter were high-occurrence seasons for disturbance, with peaks around 12:00–14:00, while the giant panda exhibited a bimodal diurnal activity pattern (10:00–12:00, 14:00–16:00), the red panda peaked mainly at 8:00–10:00, and the forest musk deer preferred crepuscular and nocturnal activity—all three species displayed activity rhythms that temporally avoided peak disturbance periods; (3) Spatially, giant pandas were sparsely distributed, red pandas showed aggregated distribution, and forest musk deer exhibited a multi-core distribution, with the core distribution areas of each species spatially segregated from high-disturbance zones; (4) GAM analysis revealed that the red panda responded most significantly to disturbance, the giant panda showed marginal significance, and the forest musk deer showed no significant response. This study systematically elucidates the spatiotemporal differences in responses to multiple human disturbances among three sympatric species within the same landscape, providing a scientific basis for the management of human activities, habitat optimization, and synergistic biodiversity conservation in protected areas. It holds practical significance for promoting harmonious coexistence between human and wildlife.

## Simple Summary

Human disturbance drives global biodiversity loss by disrupting wildlife behavior and populations, yet multi-disturbance analyses in protected areas remain scarce. We examined three sympatric species, giant panda (*Ailuropoda melanoleuca*), red panda (*Ailurus fulgens*), and forest musk deer (*Moschus berezovskii*) in Sichuan Liziping Nature Reserve via infrared cameras, spatiotemporal analysis and Generalized Additive Models (GAM). Seven disturbance types were identified, with cattle, goat and walking disturbances dominant; disturbances peaked diurnally (12:00–14:00) in summer and winter, concentrating near settlements. The three species employed distinct spatiotemporal avoidance strategies. The GAM analysis revealed that the disturbance response was strongest in red pandas, marginally significant in giant pandas, and non-significant in forest musk deer. Our findings clarify species-specific adaptation mechanisms, providing scientific support for reserve management and human–wildlife coexistence.

## 1. Introduction

Rapid population growth and the continuous expansion of human activity have led to increasing intensity and spatial extent of human disturbances. These disturbances have significantly disrupted the natural distributional balance and behavioral rhythms of wildlife worldwide [1,2]. Research indicates that human activities influence animal behavior in multidimensional ways. On one hand, habitat fragmentation and the attraction of artificial resources directly affect animal movement patterns. In areas of high human pressure, mammalian movement distances may decrease to one-half or even one-third of those observed in undisturbed habitats [1]. On the other hand, human activities can force animals to adjust their activity schedules, leading to an increased proportion of nocturnal activity on average. Species that are originally diurnal or crepuscular generally exhibit a temporal niche shift toward nighttime. This shift may narrow the activity time window and cause mismatches with natural requirements, such as creating asynchrony between foraging times and prey activity peaks, thereby emerging as a novel driver of biodiversity loss [3]. In response to disturbance, wildlife often avoid human-dominated landscapes. However, to meet metabolic energy requirements under reduced habitat quality, animals employ a variety of behavioral strategies to cope with human-induced stress and predation risk. Spatial and temporal avoidance are among the most common strategies [4,5]. Moreover, different disturbance types produce varying ecological impacts, and wildlife species differ both in how they respond to the same disturbance type and how a single species responds to different disturbances [5,6]. For instance, large herbivores often increase nocturnal activity to avoid daytime human presence [3], while small mammals may shorten foraging bouts or shift microhabitat use to mitigate sudden human intrusion [7]. Although behavioral plasticity may offer short-term survival benefits, it can incur long-term ecological costs such as energy imbalance and reduced reproductive success, ultimately leading to population declines [8,9].

The giant panda (*Ailuropoda melanoleuca*) and red panda (*Ailurus fulgens*) are iconic species endemic to the Himalayan–Hengduan Mountain region [10]. Today, wild giant panda populations are restricted to fragmented mountain ranges in Shaanxi, Gansu, and Sichuan Provinces [11]. In contrast, the red panda has a much wider distribution range, extending from central Nepal through Bhutan, India, and Myanmar into southwestern China [12]. The forest musk deer (*Moschus berezovskii*) is more broadly distributed across East Asia, though its populations are highly fragmented, with core strongholds in central and southwestern China [13]. In the subalpine forests of Sichuan, these three species form a unique sympatric assemblage. Despite occupying distinct ecological niches, they coexist within the same habitat. Specifically, niche differentiation among the three species is manifested across three primary dimensions. First, dietary niche divergence. The giant panda is a highly specialized obligate bamboo feeder, with bamboo constituting nearly its entire diet. The red panda also specializes in bamboo but exhibits omnivorous tendencies, supplementing its diet with fruits, insects, and other items [14,15]. In contrast, the forest musk deer is a typical folivorous browser, consuming young leaves, twigs of various woody plants, and herbaceous vegetation [13]. This reflects a fundamental separation in trophic resource use from the two bamboo-dependent species. Second, differentiation in spatial distribution and microhabitat. Studies in the Daxiangling Mountain area indicate that suitable habitats for giant pandas are mainly distributed in coniferous forests, broadleaf forests, and shrublands at elevations of 2300–3200 m, away from roads. Suitable habitats for red pandas are preferentially found in coniferous and mixed coniferous-broadleaf forests at elevations of 2200–3200 m. Although the total suitable habitat areas for the two species are similar and exhibit a high overlap index, the overlap index for highly suitable habitats is significantly reduced, suggesting spatial segregation in core high-quality habitats to mitigate competition [14]. The forest musk deer tends to select steep slopes with dense shrub cover and complex topography at elevations of 2000–3800 m, showing clear spatial differentiation from the other two species [13]. Third, temporal activity rhythm partitioning. Analysis of daily activity patterns indicates that giant pandas display a bimodal crepuscular pattern, with peaks around 8:00 and 18:00, whereas red panda activity is concentrated around 11:00. The daily activity overlap index between the two species is 0.87, differing significantly [14]. The forest musk deer exhibits a strongly nocturnal pattern. This contrasts sharply with its diurnal counterparts, the giant and red panda, demonstrating distinct temporal niche segregation [16]. This multidimensional niche differentiation enables the three species to effectively minimize interspecific competition and facilitate complementary resource use within shared habitats. However, the stability of this assemblage is increasingly threatened by human disturbance. Giant panda habitats are affected by the expansion of roads, farmland, and settlements, leading to severe habitat fragmentation [17]. Red pandas face direct pressure from livestock grazing and human activities [18]. Forest musk deer, historically impacted by overhunting, remain highly vulnerable [16]. Although previous studies have independently addressed the threats faced by these species, systematic comparative research examining multiple disturbance types within a single protected area remains limited.

To better understand human disturbance patterns and species responses within a shared ecosystem, this study was conducted in the Liziping National Nature Reserve. We investigated the types and spatiotemporal distribution patterns of human disturbances within the habitats of the three species in this area. We further explored their response strategies to these disturbances and the differential responses among the three sympatric study species to key disturbance types. The results provide scientific evidence for biodiversity conservation, human activity management, and habitat optimization within protected areas, ultimately contributing to the goal of promoting harmonious coexistence between humans and wildlife.

## 2. Materials and Methods

### 2.1. Study Area

**Figure 1:**
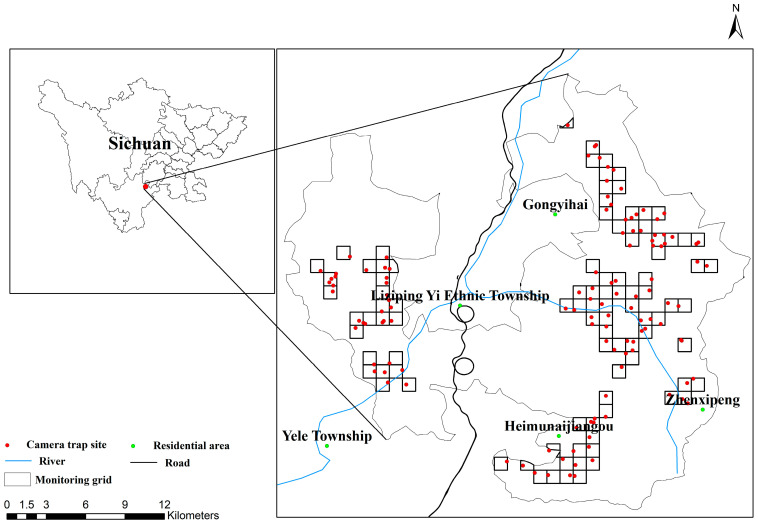
Overview map of the study area and location of the camera traps.

The Sichuan Liziping National Nature Reserve is located in the southern part of Shimian County, Ya’an City, Sichuan Province (102°11′06″–102°30′21″ E, 28°51′02″–29°08′54″ N). It spans 23 km north–south and 17.8 km east–west, covering a total area of 47,940 hm^2^ (Figure 1). The highest elevation within the reserve is 4551 m, while the lowest is 1330 m, with an elevational range of 3221 m. The reserve experiences a subtropical monsoon climate with distinct seasons: spring (March–May), summer (June–August), autumn (September–November), and winter (December–February). The annual average temperature ranges from 11.7 to 14.4 °C, with annual precipitation between 800 and 1250 mm. The reserve supports rich biodiversity and a variety of vegetation types typical of subalpine forest ecosystems. Four townships and eight villages surround the reserve, with residents primarily relying on agriculture, animal husbandry and tourism for their livelihoods.

### 2.2. Infrared Camera Deployment

Following established protocols for camera trap studies [19,20], this research was conducted from May 2022 to August 2023. The core area of the reserve was randomly divided into 1 km × 1 km grids using ArcGIS 10.8, resulting in a total of 98 monitoring grids and the installation of 124 cameras. Within each grid, infrared cameras were placed along travel routes such as mountain trails and near water sources, where wildlife, domestic animals and human activities are frequent, based on the field experience of reserve staff. Since high-activity pathways within a single grid are often distributed across different habitats and cannot be fully covered by a single camera, some grids were equipped with 2–3 cameras depending on the dispersal of pathways, in order to improve monitoring coverage and data completeness (Figure 1). The cameras used were Ltl ACORN 6511 models (Zhuhai Ltl Acorn Electronics Co., Ltd., Zhuhai, China), which were numbered and mounted on tree trunks 30–80 cm above ground level. Camera settings followed the technical guidelines for infrared camera monitoring of terrestrial vertebrates [21], configured to operate in burst mode of three still images followed by one video recording, with each video lasting 10 s and a 5 s interval between triggers.

### 2.3. Data Analysis

Analysis of camera trap data indicates that human disturbance within the Liziping Reserve can be categorized into three primary types: human, livestock and domestic dog (*Canis lupus familiaris*) disturbance. Based on the nature of the disturbance, duration and ecological impact, human disturbance was further categorized into walking and gathering activities. Simultaneously, the grazing livestock recorded within the reserve included cattle (*Bos primigenius taurus*), yak (*Bos grunniens*), and horse (*Equus caballus caballus*). A small number of sheep (Caprinae) were present in the reserve, but the vast majority were goats (Caprinae). Therefore, in this paper, the term “goat” is used to encompass both goats and sheep.

To exclude the impact of repeated short-term captures of the same target on disturbance frequency statistics, consecutive photographs of the same species taken by the same camera within 30 min were recorded as only one independent valid photograph for that species [22]. Furthermore, 24 consecutive hours of field operation for an infrared camera at a single site was considered one valid camera-day [23,24,25].

#### 2.3.1. Activity Rhythm Index Calculation

The daily and annual activity rhythms of giant pandas, red pandas, forest musk deer and various disturbance types were characterized by calculating the Time-period Relative Abundance Index (TRAI) and the Month Relative Abundance Index (MRAI) [24]. The formulas are as follows:

Time-period relative abundance index: TRAI = (T_ij_/N) × 1000

Month relative abundance index: MRAI = (M_ij_/N) × 1000

where T_ij_ represents the number of valid independent photographs of animal or disturbance type j during time interval i, M_ij_ denotes the number of valid independent photographs of animal or disturbance type j during month i, and N is the total number of valid camera operation days [26]. The RAI value for each species or disturbance type in each time period was calculated based on this index, and the 95% confidence interval (95% CI) was estimated using the Bootstrap method (1000 replicates).

#### 2.3.2. Temporal Overlap Analysis

Peak Overlap Identification. For daily activity rhythms, the peak activity periods of the target species (giant panda, red panda, forest musk deer) were used as benchmarks to identify other disturbance species or types whose active periods overlapped with these peaks. Annual activity rhythm analysis did not involve peak overlap judgment; temporal overlap was quantified solely using the overlap coefficient.

Overlap Coefficient Calculation. The daily activity rhythm overlap coefficient was calculated based on kernel density estimation. The annual activity rhythm overlap coefficient was calculated using the formula Δ = ∑min (p1i, p2i), where p represents the activity proportion in each time period or month. Both types of overlap coefficients range from 0 to 1, with higher values indicating greater temporal overlap between species or between species and disturbances. The 95% CI for both coefficients was estimated using the Bootstrap method (1000 replicates).

#### 2.3.3. Statistical Tests

Differences in daily activity rhythms were tested using the Kolmogorov–Smirnov (K-S) test. Differences in annual activity rhythms were assessed using the Chi-square test of independence.

#### 2.3.4. Modeling the Quantitative Relationship Between Species Activity Intensity and Human Disturbance

To systematically quantify the overall response patterns of giant pandas, red pandas, and forest musk deer to human disturbance, this study used infrared camera monitoring data. The Relative Activity Index (RAI)—defined as the number of independent detections per 100 camera days—served as the quantitative measure of species activity intensity. Seven types of human disturbance were combined into a composite disturbance index. A Generalized Additive Model (GAM) was constructed to analyze the nonlinear relationship between species activity and disturbance intensity. The model used a Tweedie distribution to fit the response variable, a smoothing function s() to capture potential nonlinear trends, and Restricted Maximum Likelihood (REML) for parameter estimation. To visualize the nonlinear relationships fitted by the GAMs, partial effect plots for each species were generated using the ggplot2 (version 4.0.1) and mgcv (version 1.9.3) packages in R 4.5.2, graphically revealing the response patterns of different species’ activity intensity to human disturbance. This was supplemented by Spearman’s rank correlation analysis to reveal the quantitative relationships between target species activity and human disturbance from both nonlinear and linear perspectives.

The data analyses presented in Section 2.3.1, Section 2.3.2, Section 2.3.3 and Section 2.3.4 were carried out using R software, version 4.5.2.

#### 2.3.5. Spatial Distribution Characteristics

Based on the fishnet grid generated in ArcGIS, the spatial distribution characteristics of giant pandas, red pandas, forest musk deer and various disturbance types were visualized. To quantitatively assess the spatial relationship between these three target species and human disturbance, the Spatial Overlap Index (I_O_) was employed to measure the degree of spatial utilization overlap [27]. The formula is as follows:

I_O_ = O_ij_/(O_i_× O_j_)^1/2^

where I_O_ denotes the Spatial Overlap Index; O_ij_ represents the number of grids where both the target species and human disturbance were recorded; O_i_ and O_j_ are the total numbers of grids where the target species and human disturbance were recorded, respectively. The value of I_O_ ranges from 0 to 1, with 0 indicating completely non-overlapping spatial distributions and 1 indicating perfectly coincident spatial distributions.

## 3. Results

**Figure A1:**
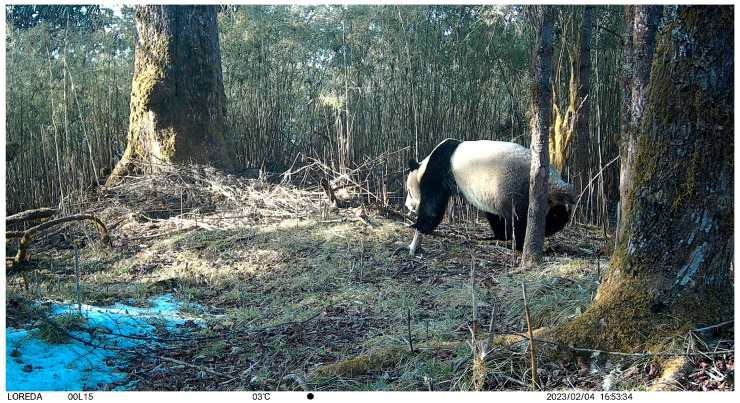
Giant panda (Photographed in Liziping Nature Reserve).

**Figure A2:**
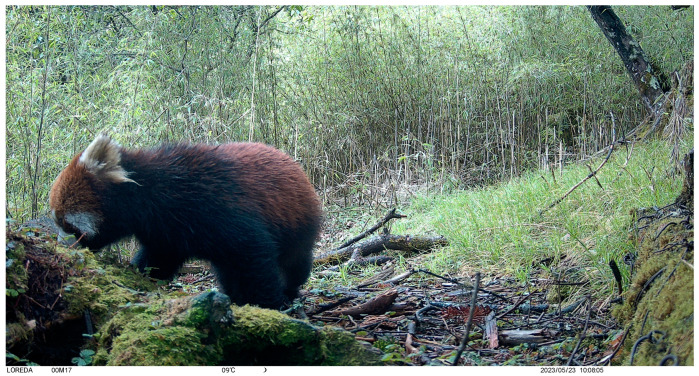
Red panda (Photographed in Liziping Nature Reserve).

**Figure A3:**
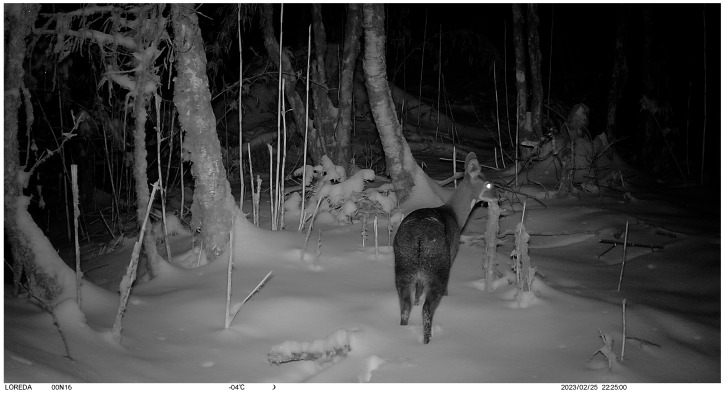
Forest musk deer (Photographed in Liziping Nature Reserve).

During the infrared camera monitoring period, we obtained 3512 effective camera-trap days and collected 41,130 valid photographs after removing invalid data due to equipment failure or environmental interference. Using the “30-min independence rule” for screening, we identified 15 valid photographs of giant pandas (Figure A1), 42 of red pandas (Figure A2) and 27 of forest musk deer (Figure A3). Additionally, 1057 valid photographs recorded human disturbance.

### 3.1. Types and Frequency of Disturbances

**Figure 2:**
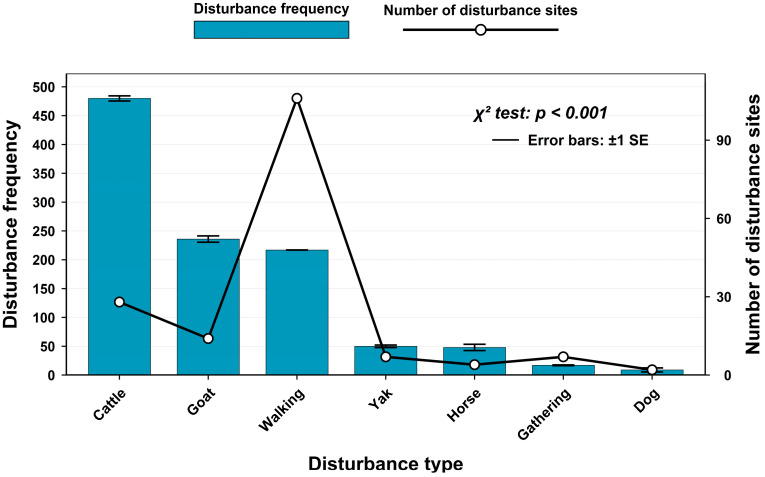
Frequency of disturbance and number of monitoring sites affected.

**Figure A4:**
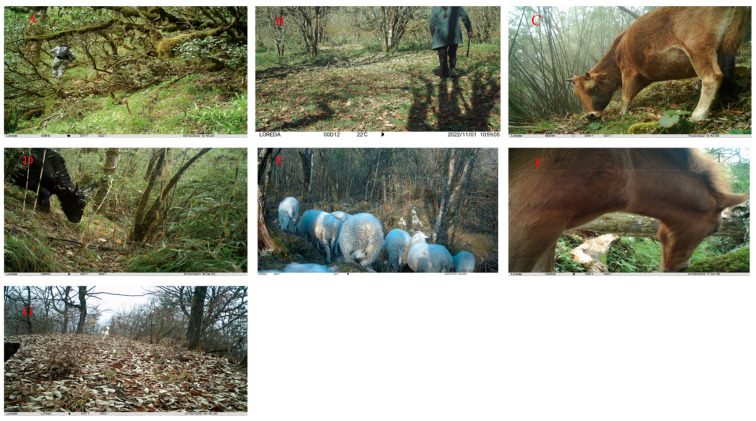
Human disturbance ((**A**): Walking; (**B**): Gathering; (**C**): Cattle; (**D**): Yak, (**E**): Goat, (**F**): Horse; (**G**): Domestic dog. Photographed in Liziping Nature Reserve).

Seven types of human disturbance were recorded within the study area during the monitoring period (Figure A4). Ranked by frequency from highest to lowest, they were: cattle disturbance (480 events), goat disturbance (236 events), walking disturbance (217 events), yak disturbance (50 events), horse disturbance (48 events), gathering disturbance (17 events) and domestic dog disturbance (9 events). Ranked by the number of monitoring sites affected, they were: walking disturbance (106 sites), cattle disturbance (28 sites), goat disturbance (14 sites), yak disturbance (7 sites), gathering disturbance (7 sites), horse disturbance (4 sites) and domestic dog disturbance (2 sites). A Chi-squared test indicated that the frequency and spatial distribution across disturbance types differed significantly (Figure 2).

### 3.2. Temporal Patterns of Disturbances and Activity Rhythms of the Giant Panda, Red Panda, and Forest Musk Deer

**Figure 3:**
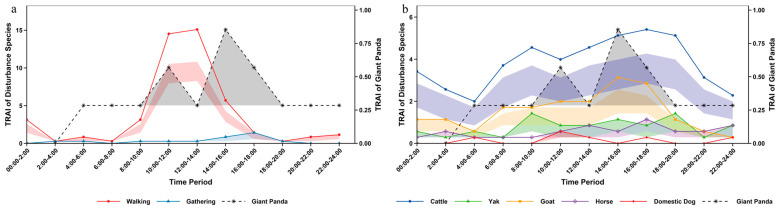
Daily activity rhythm of giant panda and human disturbance in Liziping Nature Reserve. The shaded area around the curves represents the 95% confidence intervals calculated based on 1000 Bootstrap resamples. (**a**) Daily activity rhythm of giant panda and human disturbance; (**b**) Daily activity rhythm of giant panda and disturbance from livestock and domestic dogs.

Analysis of daily activity rhythms based on infrared camera data revealed that the giant panda exhibits a diurnal bimodal pattern. Its primary activity peak occurred between 14:00 and 16:00, with a secondary peak between 10:00 and 12:00 (Figure 3). Temporal overlap and Kolmogorov–Smirnov test results indicated that the giant panda’s activity peak overlapped only with that of goats. No peak overlap was found with other livestock such as cattle, yaks, horses or domestic dogs. Furthermore, the temporal distribution differences between the giant panda and these livestock were not significant. Regarding human disturbance, the giant panda showed no peak overlap with walking or gathering disturbances. A significant difference in temporal distribution was found with walking disturbance, but not with gathering disturbance. The activity overlap coefficients between the giant panda and both types of human disturbance were very low (Table S1).

**Figure 4:**
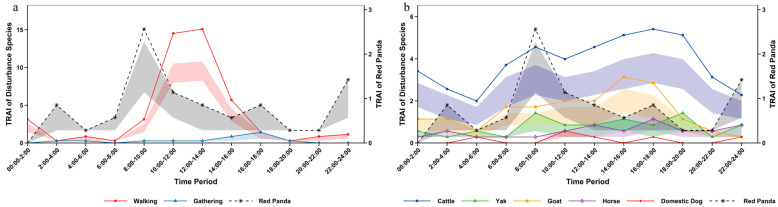
Daily activity rhythm of red panda and human disturbance in Liziping Nature Reserve. The shaded area around the curves represents the 95% confidence intervals calculated based on 1000 Bootstrap resamples. (**a**) Daily activity rhythm of red panda and human disturbance; (**b**) Daily activity rhythm of red panda and disturbance from livestock and domestic dogs.

Activity records of red pandas were obtained throughout the day, with the main peak of daily activity occurring during 8:00–10:00 (Figure 4). Comparison of temporal distribution with disturbance factors revealed that red pandas only had overlapping activity peaks with yaks (8:00–10:00), but no overlapping peaks with other livestock such as cattle, horses and domestic dogs. Moreover, a significant difference in temporal distribution was only observed between red pandas and goats, while no significant differences were detected with the remaining livestock. For human disturbance, red pandas showed no overlapping activity peaks with either walking or gathering disturbances. A significant difference existed between red pandas and walking disturbance, but no significant difference was found with gathering disturbance. The activity overlap coefficients between the two types of disturbance and red pandas were both relatively low (Table S2).

**Figure 5:**
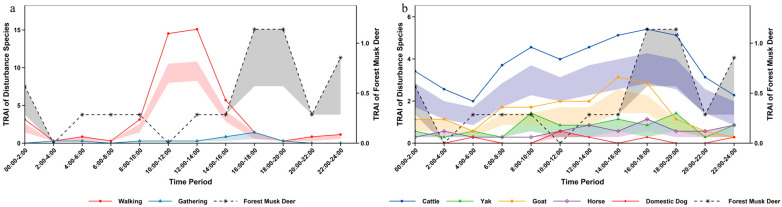
Daily activity rhythm of forest musk deer and human disturbance in Liziping Nature Reserve. The shaded area around the curves represents the 95% confidence intervals calculated based on 1000 Bootstrap resamples. (**a**) Daily activity rhythm of forest musk deer and human disturbance; (**b**) Daily activity rhythm of forest musk deer and disturbance from livestock and domestic dogs.

The activity rhythm of forest musk deer showed crepuscular and nocturnal preferences, with an overall unimodal pattern. The core peak activity period was 16:00–18:00, while the activity trough periods were 2:00–4:00 and 10:00–12:00 (Figure 5). Temporal distribution test results demonstrated that the activity overlap between forest musk deer and human disturbances (walking and gathering) was extremely low, with overlap coefficients of 0.024 and 0.041, respectively, and the differences both reached a significant level. In comparison with livestock, significant differences in temporal distribution were found between forest musk deer and cattle, yaks as well as goats, but no significant differences were observed with horses and domestic dogs. The activity time overlap coefficients between some livestock and forest musk deer were relatively high (Table S3).

From the perspective of overall rhythm, human disturbances in the reserve were mainly concentrated in the period of 8:00–18:00, while the nighttime disturbance intensity decreased significantly and tended to be stable (Table 1). Among human disturbances, the peak period of walking disturbance was 12:00–14:00, which was the type with the highest disturbance intensity throughout the day; the peak period of gathering disturbance appeared at 16:00–18:00. For livestock activities, the peak periods of both cattle and horses were 16:00–18:00, with horses showing relatively low overall disturbance intensity and stable rhythm; the peak of yaks was concentrated in 08:00–10:00; goat disturbance mainly occurred during the daytime and decreased significantly at night, with the peak period at 14:00–16:00; domestic dogs only caused slight disturbance in the early morning (4:00–6:00), noon (10:00–12:00) and afternoon (16:00–18:00), and no relevant activities were detected in other periods (Figure 3).

**Figure 6:**
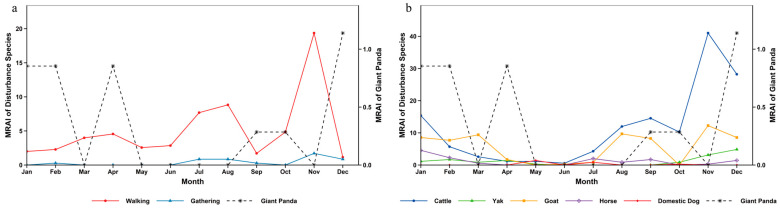
Annual activity rhythm of giant panda and human disturbance in Liziping Nature Reserve. (**a**) Annual activity rhythm of giant panda and human disturbance; (**b**) Annual activity rhythm of giant panda and disturbance from livestock and domestic dogs.

On an annual scale, the activity of giant pandas maintained relatively high levels from January to April. After April, their activity continued to decline, reaching a trough in September. Starting from October, the relative abundance gradually increased, and the activity peaked in December, during which period giant pandas were relatively active (Figure 6). Chi-square test and overlap analysis results showed that there were significant differences in temporal distribution between giant pandas and most disturbance factors. Only the temporal distribution between giant pandas and yaks showed no significant difference, with a relatively high annual overlap coefficient (Table S4).

**Figure 7:**
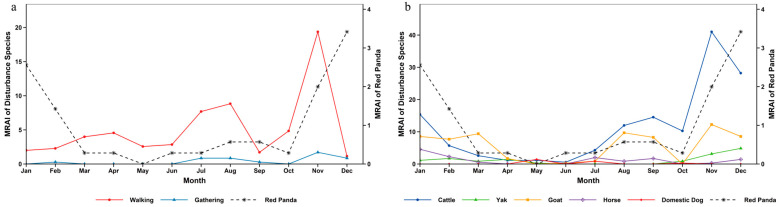
Annual activity rhythm of red panda and human disturbance in Liziping Nature Reserve. (**a**) Annual activity rhythm of red panda and human disturbance; (**b**) Annual activity rhythm of red panda and disturbance from livestock and domestic dogs.

The annual activity rhythm of red pandas exhibited characteristics of phased variation: the activity level was relatively high in January, then continued to decline and remained low in March; the activity level stayed stable from March to July; it began to rise gradually from July, forming a minor activity peak between July and October; after October, the activity level kept increasing and reached the annual peak in December (Figure 7). Temporal overlap and significance analysis indicated that there were significant differences in temporal distribution between red pandas and walking disturbance, goats, horses as well as domestic dogs, among which the annual overlap coefficient between red pandas and domestic dogs was the lowest. In contrast, no significant differences were found between red pandas and gathering disturbance, cattle or yaks, with relatively high annual overlap coefficients—and the overlap degree with yaks was the highest (Table S5).

**Figure 8:**
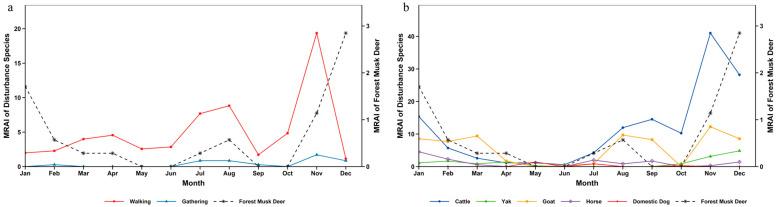
Annual activity rhythm of forest musk deer and human disturbance in Liziping Nature Reserve. (**a**) Annual activity rhythm of forest musk deer and human disturbance; (**b**) Annual activity rhythm of forest musk deer and disturbance from livestock and domestic dogs.

Similar to the annual activity rhythm of red pandas, the activity of forest musk deer was relatively high in January, then decreased continuously and remained at a low level in March. From March to July, the activity level stayed stable within a low-level range. Starting from July, the activity level rose gradually and peaked in December across the whole year (Figure 8). Test results demonstrated that the differences between forest musk deer and walking disturbance, goats, horses as well as domestic dogs reached a significant level, while no significant differences were observed between forest musk deer and gathering disturbance, cattle or yaks (Table S6).

The peak seasons of human disturbance in the reserve were summer and winter (Table 2). The monthly relative abundance of walking disturbance presented multi-stage dynamic changes: a minor peak occurred from February to May, the first major peak appeared in August, it dropped to the annual lowest in September, and declined after the second peak emerged in November. The overall level of gathering disturbance was low with small seasonal fluctuation amplitude, concentrating mainly in summer and autumn. Among livestock disturbances, the monthly relative abundance of cattle showed phased fluctuation, falling to the lowest in June and peaking in November across the year. The overall intensity of yak disturbance remained stable, rising slightly from October and reaching the highest value in December. Goat disturbance exhibited obvious seasonality, with a minor peak in March, a recovery after July and the annual peak in November. Horse disturbance stayed at a low level for a long time and dropped to the annual minimum in November. Domestic dog disturbance had significant temporal limitation, with only a small number of disturbance records detected in May, July and October, and no obvious disturbance monitored in other months (Figure 6).

### 3.3. Spatial Patterns of Disturbances and Spatial Distribution Characteristics of the Giant Panda, Red Panda, and Forest Musk Deer

**Figure 9:**
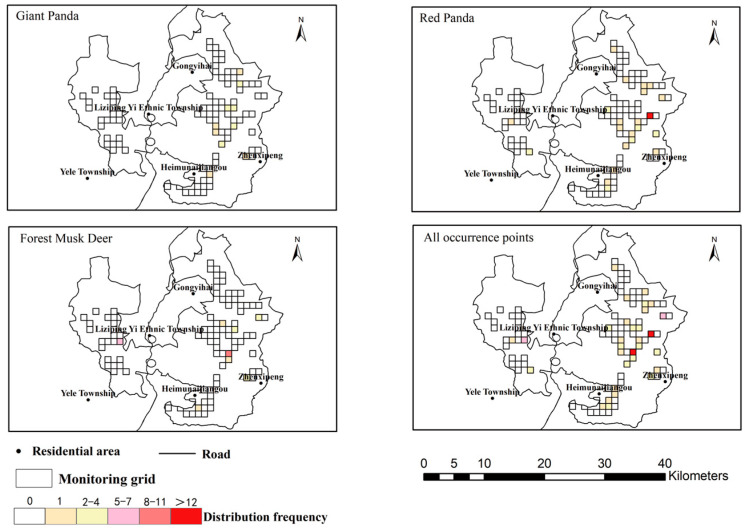
Distribution of the giant panda, red panda, and forest musk deer in the Liziping Nature Reserve. In the distribution map of a single species, the color represents the occurrence frequency of the species in the corresponding grid cell and in the schematic diagram of all occurrence sites, the color represents the absolute value of the species’ occurrence frequency in the grid cell.

The giant panda had a relatively wide but sparse distribution range within the reserve, mainly concentrated in areas such as Gongyihai and Heimunaijiangou. The red panda exhibited a spatially aggregated distribution pattern, with one high-frequency distribution point located east of Liziping Yi Township and medium-to-low frequency distribution in other areas. The forest musk deer showed a multi-core distribution pattern, occurring both near Yele Township in the west and around Heimunaijiangou in the central-eastern part of the reserve (Figure 9).

**Figure 10:**
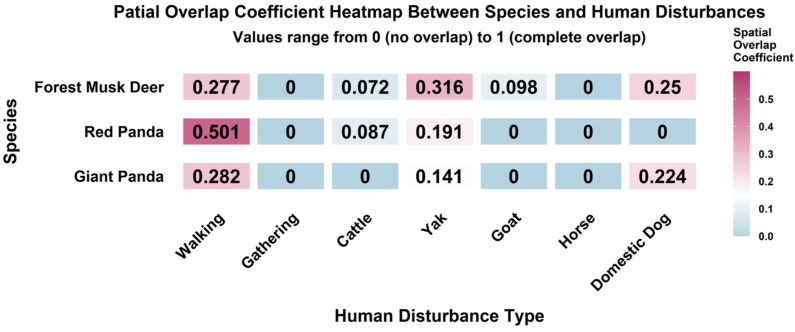
Spatial overlap coefficient heatmap between species and human disturbances.

**Figure 11:**
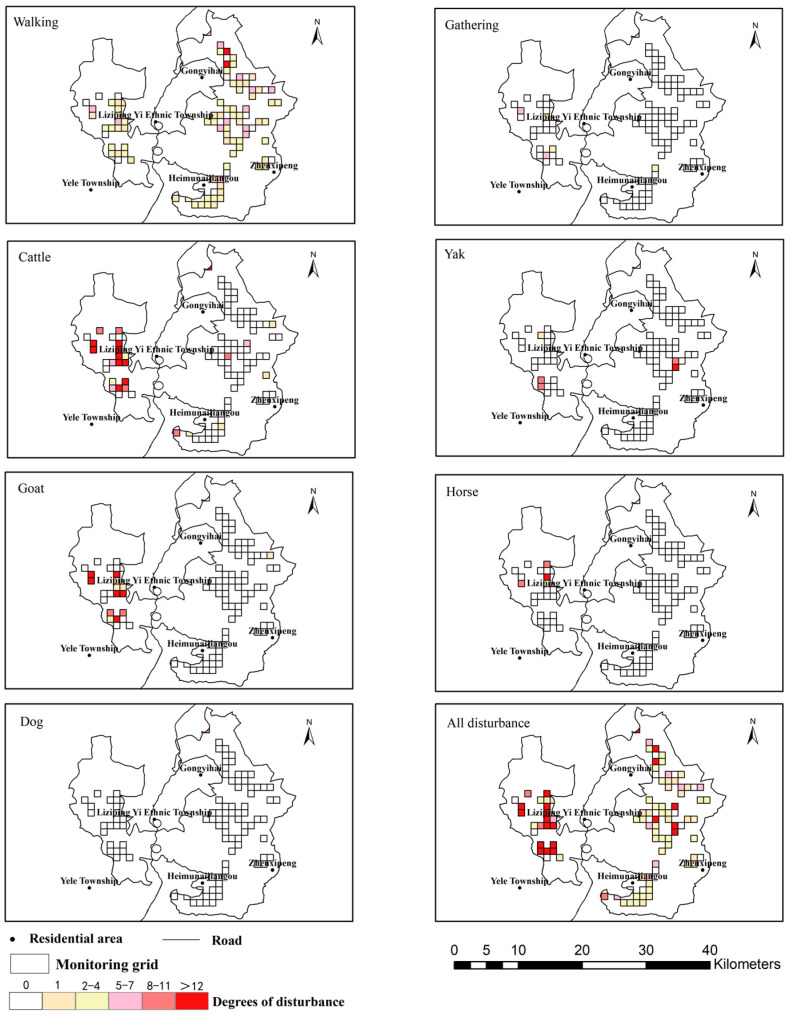
Distribution of different disturbance types in the Liziping Nature Reserve. In the distribution map of a single disturbance type, the color represents the occurrence frequency of disturbances in the corresponding grid cell and in the schematic diagram of all disturbances, the color represents the absolute value of the disturbance frequency in the grid cell.

Human disturbance had the widest distribution among all disturbance types in Liziping Nature Reserve, with disturbance photos recorded at 107 monitoring sites. Specifically, walking disturbance was mainly concentrated in Gongyihai, Liziping Yi Ethnic Township and the surrounding areas of Heimunaijiangou. It had the highest spatial overlap coefficient with red pandas (0.501), followed by that with giant pandas (0.282) and forest musk deer (0.277). Gathering disturbance occurred near residential areas in Liziping Yi Ethnic Township and Yele Township, but its spatial overlap coefficients with the three species were all 0. Among livestock disturbances, cattle disturbance had the largest distribution range, showing weak spatial overlap with red pandas (0.087) and forest musk deer (0.072), but no overlap with giant pandas. Goat disturbance ranked second in distribution range, with high overlap with the high-intensity areas of cattle disturbance; both were mainly concentrated in Liziping Yi Ethnic Township and Yele Township, and only showed weak overlap with forest musk deer (0.098). Yak disturbance was concentrated near Yele Township, with the highest overlap coefficient with forest musk deer (0.316), and relatively low overlap with red pandas (0.191) and giant pandas (0.141). Horse disturbance occurred near Liziping Yi Ethnic Township, with overlap coefficients of 0 with all three species. Domestic dog disturbance was only recorded in areas around Gongyihai and Zhenxipeng, showing a certain degree of overlap with forest musk deer (0.25) and giant pandas (0.224), but no overlap with red pandas (Figure 10 and Figure 11).

After overlaying all disturbance types, the high-disturbance areas were mainly concentrated in Gongyihai, the western part of the reserve (near Yele Township) and some areas of Liziping Yi Ethnic Township—these were the superimposed areas of multiple disturbances such as walking, cattle and goat disturbances. The medium-disturbance areas had a wide distribution, covering regions such as the periphery of Liziping Yi Ethnic Township. The non-disturbance or low-disturbance areas were concentrated in the eastern and northern parts of the reserve, which were far away from residential areas (Figure 11).

### 3.4. Quantitative Analysis of the Relationship Between Species Activity Intensity and Human Disturbance

This study adopted the Generalized Additive Model (GAM) to analyze the relationship between the activity intensity of forest musk deer, giant pandas, red pandas and human disturbance. The model fitting results showed that there were significant differences in the response degrees of different species to human disturbance (Table 3). Among them, the GAM model for red pandas had the optimal explanatory power, followed by that for giant pandas, while the response of forest musk deer was not significant.

**Figure 12:**
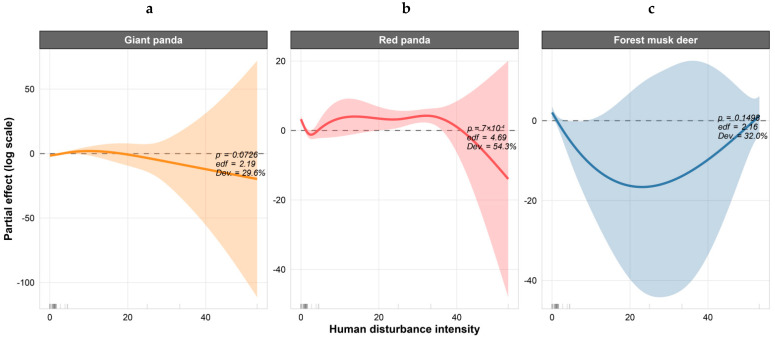
Partial Effect Plots of the Generalized Additive Model (GAM). (**a**) Partial effect plot of GAM for Giant panda; (**b**) Partial effect plot of GAM for Red panda; (**c**) Partial effect plot of GAM for Forest musk deer.

For giant pandas, the GAM model showed marginal significance, with a deviance explained of 29.60%, an effective degrees of freedom (edf) of 2.19, and an adjusted R^2^ of 0.307 (Table 3). The partial effect plot revealed that the partial effect of giant panda activity intensity first increased slightly and then declined slowly with increasing human disturbance intensity. However, the wide confidence intervals that consistently encompassed the zero-effect line indicate that human disturbance only had limited explanatory power for its activity and did not reach statistical significance (Figure 12a). For red pandas, the GAM model demonstrated high statistical significance, with a deviance explained of 54.30%, an edf of 4.69, and an adjusted R^2^ of 0.343 (Table 3). The partial effect plot indicated a pronounced nonlinear trend in the partial effect of red panda activity intensity, showing an initial decline, followed by an increase, and then a rapid decrease. The edf close to 5 further confirms this complex nonlinear response pattern (Figure 12b). For forest musk deer, the GAM model did not reach statistical significance, with a deviance explained of 32.0%, an edf of 2.16, and an adjusted R^2^ of only 0.069 (Table 3). The partial effect plot showed that the partial effect of its activity intensity first decreased and then recovered with increasing disturbance intensity, but the overall fluctuation was minimal, and the confidence intervals were very wide, reflecting the weak explanatory power of the model for the activity of forest musk deer (Figure 12c).

**Figure 13:**
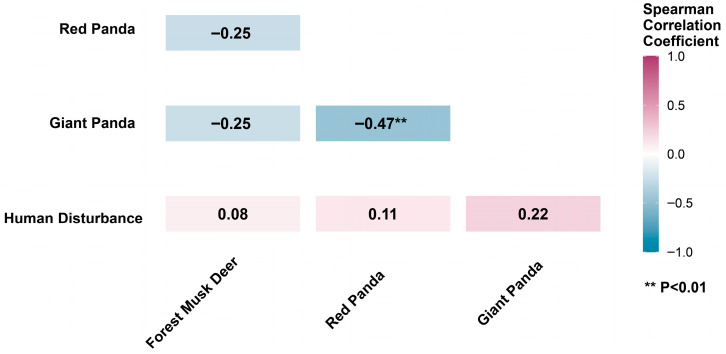
Correlation Heatmap of Species Activity Intensity and Human Disturbance Levels.

Corresponding Spearman analysis results were consistent with the GAM model trends. The activities of both giant pandas and red pandas showed positive correlations with human disturbance, whereas forest musk deer exhibited only a very weak positive correlation with human disturbance (r = 0.08), which is consistent with the low explanatory power of the model (Figure 13).

## 4. Discussion

### 4.1. Differences in Ecological Effects Among Disturbance Types

Through monitoring human disturbances in Sichuan Liziping National Nature Reserve, this study identified a total of 7 types of human disturbances. Chi-square test results showed that there were extremely significant differences in the frequency and site distribution of different disturbance types. This differentiation in frequency and spatial distribution directly led to significant variations in the ecological effects of different disturbance types on giant pandas, red pandas and forest musk deer. The impact pathways and intensities depended on the matching degree between the spatiotemporal characteristics of disturbances and the ecological habits of the species. As the most widely distributed disturbance type, walking disturbance is a direct intrusion of human activities. Its daily activity peak was concentrated in the period of 12:00–14:00, and overall disturbances mainly occurred during 8:00–18:00, the active hours of human activities. From the perspective of species responses, giant pandas showed obvious spatial avoidance characteristics to such disturbances, with a spatial overlap coefficient of only 0.282. In addition, there was a significant difference in the diurnal activity time distribution between giant pandas and walking disturbance, with no peak overlap, reflecting the direct occupation effect of such disturbances on giant panda habitats. Red pandas had the highest spatial overlap coefficient with walking disturbance (0.501), but there was a significant difference in temporal distribution with no peak overlap, suggesting that they might avoid disturbances by adjusting their activity timing. For the forest musk deer, its extremely low spatial overlap coefficient with hiking disturbance (0.024) indicates a significant compression effect of the disturbance on its activity space, while the significant difference in temporal distribution is more likely related to the forest musk deer’s innate nocturnal activity rhythm. Such disturbances alter microhabitat vegetation structure through frequent trampling and trigger the “landscape of fear” effect [5], resulting in the reduction of activity ranges of wild animals in human-dominated areas. This is a pattern observed globally [1], especially evident in the avoidance behavior of medium and large mammals towards recreational trails [28]. The differences in spatiotemporal overlap between different species and walking disturbance in this study also reflect the differentiation of their adaptation strategies to disturbances.

Livestock disturbance affects habitats mainly through two pathways: resource competition and physical damage, and there are obvious differences in the disturbance characteristics and ecological effects of different livestock species. The results showed that cattle disturbance had the highest frequency and was spatially concentrated in the western part of the reserve. It had weak spatial overlap with red pandas and forest musk deer, but no overlap with giant pandas. Its daily activity peak was 16:00–18:00, with no significant difference in temporal distribution from giant pandas and red pandas, but no peak overlap, implying potential resource competition pressure under long-term coexistence. Sharma et al. found in their study on the relationship between livestock and red pandas that cattle directly consume bamboo resources [18], which poses a direct threat to giant pandas and red pandas that mainly feed on bamboo. Moreover, soil structure damage caused by frequent trampling further accelerates habitat degradation [29]. In contrast, the disturbance frequency of yaks, goats, and horses was relatively low, but their large-scale mobile activity characteristics may cause fragmentation effects on local habitats such as shrubs that forest musk deer rely on.

In this study, although the frequency of gathering and domestic dog disturbances was low, their “potential threat” characteristics pose hidden and irreversible ecological risks to specific species. Gathering disturbance was mainly concentrated in summer and autumn, with a peak period of 16:00–18:00. Spatially, it occurred near residential areas in Liziping Yi Ethnic Township and Yele Township, but the spatial overlap coefficients with the three species were all 0. Temporally, there was no significant difference in distribution between giant pandas and red pandas, but the overlap coefficients were extremely low, while the overlap coefficient with forest musk deer was only 0.041, with a significant temporal difference. Activities such as gathering bamboo shoots and medicinal herbs involve digging, cutting down or disturbing understory vegetation. They directly damage the vertical structure of forests, causing changes in microenvironments like light and humidity inside the forests. This dual impact of structural loss and direct resource depletion weakens the integrity of dense bamboo thickets and tree holes that giant pandas need for raising cubs and hiding [30]. For red pandas, it directly endangers the mature trees (used as nests and shelters) and complex understory structures they depend on for survival [10]. Domestic dog disturbance has temporal and spatial limitations, but it poses direct predation pressure on medium and small ungulates such as forest musk deer. As potential carriers of zoonotic diseases (e.g., canine distemper, rabies), domestic dogs also pose a severe long-term survival threat to susceptible populations such as giant pandas [31]. The “low-frequency but high-intensity” nature of such disturbances makes their ecological effects easy to underestimate, and they must be given key attention in the active management of the reserve.

### 4.2. Impact of Disturbance Temporal Dynamics on Species Activity Rhythms

Species activity rhythms are the result of long-term environmental adaptation. When the temporal dynamics of human disturbances overlap with a species’ inherent rhythms, significant ecological impacts on its survival and reproduction can occur [3]. In this study, such effects may be further exacerbated during winter when disturbances overlap with peak periods of species activity. On a monthly scale, this study found that the relative abundance of walking and livestock disturbances peaked from November to December, coinciding with an increasing trend in the relative abundance of giant panda, red panda, and forest musk deer. In winter, except for some cold-tolerant livestock (e.g., yaks), most livestock are herded back to lower-altitude villages for winter housing after November. To cope with declining food resource quality and the high energy demands of thermoregulation, giant pandas, red pandas and forest musk deer may be forced to forage in areas where disturbance intensity remains high [32,33,34]. Therefore, the temporal synchrony between such disturbances and species activities may exacerbate the negative impacts of human disturbances on species. Furthermore, generalized additive model (GAM) analysis further quantified and verified the species-specificity of such impacts. The model for red pandas exhibited the best explanatory power. The relationship between their activities and human disturbance was not a simple linear one, and the response pattern was more complex. This hints that their adaptation strategies are more diverse under the pressure of winter disturbances. The model for giant pandas showed marginal significance, with a weak positive correlation with disturbance (r = 0.22). Human disturbance had a certain explanatory power for their activities in disturbed areas during winter, but the level was not significant, which might be related to their passive foraging choices. The model for forest musk deer was not statistically significant and an extremely weak positive correlation with disturbance (r = 0.08), indicating low explanatory power. This might be due to their crepuscular and nocturnal activity habits, which reduced the overlap with concentrated diurnal disturbances in winter. This temporal synchronicity between disturbance and species activity may exacerbate the negative impacts: on one hand, winter is a critical foraging period for giant panda and a preparation period for reproduction in red panda and forest musk deer [32,35,36]. High-intensity human and livestock disturbances may compress their foraging ranges, reduce energy acquisition efficiency, and subsequently affect reproductive success. On the other hand, seasonal peaks in disturbance may disrupt species’ temporal partitioning strategies for resources, forcing adjustments in activity patterns [3], which could lead to energy metabolism imbalances over the long term [37].

Analyzed on a daily scale, the giant panda exhibited a diurnal bimodal activity pattern, consistent with the findings of Schaller et al. in Wolong National Nature Reserve [38]. The red panda was primarily diurnal but also active at night, aligning with results from Zhang et al. on wild red panda circadian rhythms in Fengtongzhai Nature Reserve [39]. The forest musk deer displayed a unimodal daily activity rhythm, matching the findings of Yang et al. in Gongga Mountain National Nature Reserve [40]. However, the peak periods for human walking and livestock activity significantly overlapped with the diurnal activity peaks of species like the red panda and forest musk deer. For the red panda, which relies on diurnal activity to forage for bamboo shoots and fruits [41], human intrusion during peak disturbance hours may reduce foraging efficiency, thereby limiting energy intake. The forest musk deer, a species with some nocturnal tendencies but also diurnal activity [16], may shift its activity rhythm towards nighttime due to daytime suppression by disturbances. In contrast, although giant pandas are crepuscular animals [42] with low flexibility of activity rhythm [43], they have avoided direct impacts to a certain extent through temporal segregation from most disturbances.

The temporal patterns of disturbance in the habitats of giant panda, red panda and forest musk deer in Liziping Reserve clearly reveal an active adaptation strategy by wildlife: temporal avoidance. From a temporal perspective, human walking disturbance and livestock disturbance exhibited significant rhythmicity: on a monthly scale, they peaked in November; on a daily scale, they concentrated in the active period of human activities from 8:00 to 18:00. Whereas the relative abundance and activity peaks of the three animal species all exhibited temporal segregation from this disturbance rhythm: giant pandas reached their activity peak in December when the disturbance intensity decreased; red pandas and forest musk deer tended to be active during periods with low disturbance intensity, such as early morning, dusk or night; and the nocturnal preference of forest musk deer was particularly pronounced. This adjustment is essentially a behavioral compromise under disturbance pressure [3]. Human walking, as the most regular disturbance factor, directly compresses the temporal niche of animals. Livestock disturbances and human activity, which overlap spatiotemporally, create a double squeeze, further forcing giant panda, red panda, and forest musk deer to intensify rhythm adjustments. In this study, gathering and dog disturbance, with their low and stable intensity, had limited driving effect on animal behavior. Walking and livestock disturbances emerged as the core factors shaping wildlife activity rhythms.

### 4.3. Association Between Species Habitat Distribution and Spatial Patterns of Human Disturbance

The grid-based analysis in this study revealed that the spatial distributions of the three target species all exhibited selective responses to human disturbances, and the strength of this response was highly correlated with the degree of ecological niche specialization. Giant pandas had a relatively wide distribution range in the reserve, but their distribution points were relatively sparse, mainly concentrated in areas such as Gongyihai and Heimainaijian Gou, showing a distinct characteristic of low-disturbance aggregation. Their core activity areas showed almost no spatial overlap with the high-frequency human walking disturbance and livestock disturbance areas. The spatial overlap coefficient with walking disturbance was only 0.282, and the overlap coefficients with cattle and goat disturbances were both 0. This pattern is directly related to the giant panda’s stringent requirement for habitat integrity—as a typical bamboo-dependent species, its foraging and reproduction require continuous, undisturbed bamboo forest ecosystems [38]. Livestock grazing-induced reduction in bamboo biomass and habitat fragmentation caused by human walking force the population to contract into areas below a certain disturbance threshold [18,44]. High-disturbance areas in the reserve were mainly concentrated in Gongyihai, the western area adjacent to Yele Township, and some parts of Liziping Yi Ethnic Township. In contrast, the core distribution areas of giant pandas were far away from these zones with overlapping multiple disturbances, which further confirms their spatial avoidance strategy against high-disturbance environments.

Although the high-frequency distribution point of red pandas, located on the east side of Liziping Yi Ethnic Township, avoided the western area with intensive livestock disturbance, it exhibited a certain degree of overlap with areas subject to low-to-moderate-intensity human walking disturbance, with the spatial overlap coefficient 0.501. This seemingly contradictory phenomenon—the contrast between the relatively high spatial overlap and the high sensitivity of red pandas to disturbances as revealed by the Generalized Additive Model (GAM)—precisely reflects the strategic flexibility of red pandas in responding to anthropogenic disturbances. Their arboreal habits and reliance on resources such as understory shrubs and bamboo shoots may render them less directly sensitive to ground-level walking disturbances compared to giant pandas [10,15,45], but this does not diminish their stringent requirements for habitat structure. This study found that disturbances in their core distribution areas were primarily occasional walking events, and the surrounding areas retained a complete vertical structure of trees, shrubs, and herbaceous layers. This supports the view of Bista et al. (2023) that red pandas can avoid immediate risks by adjusting their use of vertical space in areas with low to moderate disturbances but intact vegetation structure [46]. Therefore, a high spatial overlap coefficient does not indicate insensitivity to disturbances; rather, it suggests that their tolerance strictly depends on the “vegetation cover threshold” not being exceeded. Once disturbance intensity or frequency increases and leads to vegetation degradation, the complex nonlinear responses revealed by the GAM model may manifest as strong spatial avoidance. In addition, the spatial overlap coefficient between red pandas and yaks was 0.191. There were no significant temporal differences between red pandas and gathering or cattle disturbances, with relatively high overlap coefficients overall. This further indicates that red pandas possess a certain behavioral buffering capacity against non-persistent, low-to-moderate-intensity disturbances, but this capacity also hinges on the preservation of key vegetation structures. In contrast, the polycentric distribution pattern of forest musk deer reflects a stronger adaptability to disturbances. Their distribution sites near Yele Township in the west and around Heimunaijiangou in the mid-east correspond to areas of low-to-moderate-intensity livestock disturbance and walking disturbance, respectively. This is closely related to their omnivorous diet and broad-spectrum microhabitat requirements [47]. Forest musk deer do not rely on a single vegetation type. They can maintain their populations in moderate-to-low-intensity disturbance areas by selecting fragmented suitable microhabitats. Moreover, their crepuscular and nocturnal activity habits reduce direct contact with intensive diurnal disturbances, further enhancing their disturbance adaptability.

### 4.4. Study Limitations

During the monitoring period of this study, the number of valid samples of target species was insufficient. The valid photo counts of giant pandas, red pandas and forest musk deer were only 15, 42 and 27, respectively, with an overall small sample size and uneven distribution across species. The limited sample size made it difficult to fully capture the actual activity patterns and spatiotemporal variation characteristics of the species. This directly restricted the fitting performance of the generalized additive model (GAM) due to insufficient data support.

## 5. Conclusions

This study employed camera-trapping and spatiotemporal pattern analysis to characterize human disturbances and the corresponding behavioral responses of a sympatric assemblage comprising the giant panda, red panda, and forest musk deer in the Sichuan Liziping National Nature Reserve. A total of seven disturbance types were identified, with cattle disturbance (480 events), goat disturbance (236 events) and walking disturbance from staff patrols (217 events) constituting the dominant forms. Spatially, high-disturbance areas were concentrated in core zones close to human settlements, such as Gongyihai, Yele Township, and Liziping Yi Township. Temporally, disturbances peaked between 12:00 and 14:00, resulting in a partial overlap with the activity rhythms of the three focal species. The spatial intersection between high-disturbance zones and key habitats of the species significantly compromised habitat integrity and resource availability.

Based on the above research findings, targeted conservation management measures are required to achieve sustainable protection of this symbiotic system and precise control of human disturbances. For disturbance caused by staff patrols, optimize patrol route planning by designating ecological patrol trails. Strictly define patrol scope, frequency, and routes, prioritizing avoidance of core activity areas and time periods for giant pandas, red pandas and forest musk deer. Implement smart patrol monitoring equipment to standardize and precisely manage patrol activities, preventing frequent habitat disturbance from unregulated patrols. Regarding cattle disturbance, management should shift focus to community livestock farming transformation. Through policy guidance and compensation, surrounding communities should transition from traditional free-range grazing to concentrated pen-rearing and artificial forage bases. This eliminates livestock entry into protected areas, cutting off cattle access routes at the source and preventing trampling of habitat vegetation and resource competition. For minor disturbances like gathering and dogs, containment should be achieved through regular community outreach and routine patrols.

## Figures and Tables

**Table 1:** Daily disturbance frequency statistics of human disturbance in Liziping Nature Reserve.

Time Period	Number of Disturbance Types	Disturbance Frequency
00:00–02:00	5	61
02:00–04:00	6	35
04:00–06:00	7	27
06:00–08:00	5	57
08:00–10:00	6	106
10:00–12:00	7	165
12:00–14:00	7	180
14:00–16:00	6	132
16:00–18:00	7	133
18:00–20:00	6	76
20:00–22:00	5	42
22:00–24:00	6	43

**Table 2:** Monthly disturbance frequency statistics of human disturbance in Liziping Nature Reserve.

Months	Number of Disturbance Types	Disturbance Frequency
January	5	111
February	6	70
March	5	61
April	4	31
May	4	19
June	3	13
July	6	58
August	5	113
September	5	93
October	4	57
November	6	273
December	6	158

**Table 3:** Summary Results of Generalized Additive Models (GAM) for Three Species in Relation to Human Disturbances.

Species	*p*-Value	Significance	Effective Degrees of Freedom (edf)	Deviance Explained	Adjusted R^2^	Sample Size	Tweedie_p
Giant Panda	0.0726	ns	2.19	29.60%	0.307	34	1.069
Red Panda	7 × 10^−4^	***	4.69	54.30%	0.343	34	1.692
Forest Musk Deer	0.1498	ns	2.16	32.00%	0.069	34	1.659

ns: no significance; *** *p* < 0.001.

## Data Availability

Data will be made available upon request.

## Supplementary Materials

The following supporting information can be downloaded at: https://www.mdpi.com/article/10.3390/biology15020194/s1, Table S1: Statistical Analysis of Peak Characteristics and Overlap in Daily Activity Rhythms of Giant Panda and Human Disturbances; Table S2: Statistical Analysis of Peak Characteristics and Overlap in Daily Activity Rhythms of Red Panda and Human Disturbances; Table S3: Statistical Analysis of Peak Characteristics and Overlap in Daily Activity Rhythms of Forest Musk Deer and Human Disturbances; Table S4: Statistical Analysis of Overlap Coefficients and Chi-Square Test Results in Annual Activity Rhythms of Giant Panda and Human Disturbances; Table S5: Statistical Analysis of Overlap Coefficients and Chi-Square Test Results in Annual Activity Rhythms of Red Panda and Human Disturbances; Table S6: Statistical Analysis of Overlap Coefficients and Chi-Square Test Results in Annual Activity Rhythms of Forest Musk Deer and Human Disturbances.

## Abbreviations

The following abbreviations are used in this manuscript:

TRAI	Time-period relative abundance index
MRAI	Month relative abundance index

## Appendix A

The following are images of the target species and seven types of human disturbance:

## References

[1] Tucker M., Böhning-Gaese K., Fagan W., Fryxell J., Van Moorter B., Alberts S., Ali A., Allen A., Attias N., Avgar T. (2018). Moving in the Anthropocene: Global reductions in terrestrial mammalian movements. Science.

[2] Dirzo R., Young H., Galetti M., Ceballos G., Isaac N., Collen B. (2014). Defaunation in the Anthropocene. Science.

[3] Gaynor K., Hojnowski C., Carter N., Brashares J. (2018). The influence of human disturbance on wildlife nocturnality. Science.

[4] Mills K. (2023). Apex Predators in the Anthropocene: African Large Carnivore Ecology at the Human-Wildlife Interface. Ph.D. Thesis.

[5] Frid A., Dill L. (2002). Human-caused Disturbance Stimuli as a Form of Predation Risk. Conserv. Ecol..

[6] Stankowich T. (2008). Ungulate flight responses to human disturbance: A review and meta-analysis. Biol. Conserv..

[7] Suraci J.P., Clinchy M., Zanette L.Y., Wilmers C.C. (2019). Fear of humans as apex predators has landscape-scale impacts from mountain lions to mice. Ecol. Lett..

[8] Creel S., Christianson D. (2008). Relationships between direct predation and risk effects. Trends Ecol. Evol..

[9] Zanette L.Y., White A.F., Allen M.C., Clinchy M. (2011). Perceived predation risk reduces the number of offspring songbirds produce per year. Science.

[10] Zhang Z., Wei F., Li M., Zhang B., Liu X., Hu J. (2004). Microhabitat separation during winter among sympatric giant pandas, red pandas, and tufted deer: The effects of diet, body size, and energy metabolism. Can. J. Zool..

[11] Xu W., Viña A., Kong L., Pimm S.L., Zhang J., Yang W., Xiao Y., Zhang L., Chen X., Liu J. (2017). Reassessing the conservation status of the giant panda using remote sensing. Nat. Ecol. Evol..

[12] Glatston A., Wei F., Sherpa A. (2015). *Ailurus fulgens* (errata version published in 2017). The IUCN Red List of Threatened Species.

[13] Wang Y., Harris R. (2015). *Moschus berezovskii* (errata version published in 2016). The IUCN Red List of Threatened Species.

[14] Feng B., Bai W., Fan X., Fu M., Song X., Liu J., Qin W., Zhang J., Qi D., Hou R. (2023). Species coexistence and niche interaction between sympatric giant panda and Chinese red panda: A spatiotemporal approach. Ecol. Evol..

[15] Wei F., Feng Z., Wang Z., Li M. (1999). Feeding strategy and resource partitioning between giant and red pandas. Mammalia.

[16] Yang Q., Meng X., Xia L., Feng Z. (2003). Conservation status and causes of decline of musk deer (*Moschus* spp.) in China. Biol. Conserv..

[17] Zhao C., Yue B., Ran J., Moermond T., Hou N., Yang X., Gu X. (2017). Relationship between human disturbance and Endangered giant panda Ailuropoda melanoleuca habitat use in the Daxiangling Mountains. Oryx.

[18] Sharma H.P., Belant J.L., Swenson J.E. (2014). Effects of livestock on occurrence of the Vulnerable red panda *Ailurus fulgens* in Rara National Park, Nepal. Oryx.

[19] Burton A.C., Neilson E., Moreira D., Ladle A., Steenweg R., Fisher J.T., Bayne E., Boutin S. (2015). Wildlife camera trapping: A review and recommendations for linking surveys to ecological processes. J. Appl. Ecol..

[20] Wearn O.R., Rowcliffe J.M., Carbone C., Bernard H., Ewers R.M. (2013). Assessing the Status of Wild Felids in a Highly-Disturbed Commercial Forest Reserve in Borneo and the Implications for Camera Trap Survey Design. PLoS ONE.

[21] Rovero F., Tobler M., Sanderson J. (2010). Camera trapping for inventorying terrestrial vertebrates. Manual on Field Recording Techniques and Protocols for All Taxa Biodiversity Inventories and Monitoring.

[22] Michalski F., Peres C.A. (2007). Disturbance-mediated mammal persistence and abundance-area relationships in Amazonian forest fragments. Conserv. Biol..

[23] Yang X., Wang X.H., Fu M.X., Song X.Q., Li P., Zhang D.L., Zhou H. (2024). Activity Rhythms of Six Ungulate Species Along the Proposed Sichuan-Tibet Railway (Kangding to Batang Section) Based on Infrared Camera Surveys. J. Guizhou Norm. Univ. (Nat. Sci. Ed.).

[24] O’Brien T., Kinnaird M., Wibisono H. (2003). Crouching tigers, hidden prey: Sumatran tiger and prey populations in a tropical forest landscape. Anim. Conserv..

[25] Yasuda M. (2004). Monitoring diversity and abundance of mammals with camera traps: A case study on Mount Tsukuba, central Japan. Mammal. Study.

[26] Wen X., Cheng X., Dong Y., Wang Q., Lin X. (2020). Analysis of the activity rhythms of the great gerbil (*Rhombomys opimus*) and its predators and their correlations based on infrared camera technology. Glob. Ecol. Conserv..

[27] Pianka E.R. (1974). Niche Overlap and Diffuse Competition. Proc. Natl. Acad. Sci. USA.

[28] George S.L., Crooks K.R. (2006). Recreation and large mammal activity in an urban nature reserve. Biol. Conserv..

[29] Foley J.A., DeFries R., Asner G.P., Barford C., Bonan G., Carpenter S.R., Chapin F.S., Coe M.T., Daily G.C., Gibbs H.K. (2005). Global Consequences of Land Use. Science.

[30] Wei W., Swaisgood R.R., Owen M.A., Pilfold N.W., Han H., Hong M., Zhou H., Wei F., Nie Y., Zhang Z. (2019). The role of den quality in giant panda conservation. Biol. Conserv..

[31] Doherty T.S., Dickman C.R., Glen A.S., Newsome T.M., Nimmo D.G., Ritchie E.G., Vanak A.T., Wirsing A.J. (2017). The global impacts of domestic dogs on threatened vertebrates. Biol. Conserv..

[32] Zhang J., Hull V., Huang J., Zhou S., Xu W., Yang H., McConnell W.J., Li R., Liu D., Huang Y. (2015). Activity patterns of the giant panda (*Ailuropoda melanoleuca*). J. Mammal..

[33] Zhang Z.J. (2005). Study on Microhabitat Selection and Activity Patterns of Red Pandas (*Ailurus fulgens*) in Fengtongzhai. Ph.D. Thesis.

[34] Li P., Zhang Z., Yang H., Wei W., Zhou H., Hong M., Fu M., Song X., Yu J. (2021). Study on the Activity Rhythms of Ungulates in DaXiangLing Nature Reserve Based on Infrared Camera Trapping. J. Sichuan For. Sci. Technol..

[35] Wei F., Feng Z., Wang Z., Hu J. (1999). Current distribution, status and conservation of wild red pandas *Ailurus fulgens* in China. Biol. Conserv..

[36] Li Y., Shi M., Zhang B., Wu J., Wang Y., Li M., Wu Y., Hu X., Hu D., Huang Z. (2022). Effects of different weaning times on the stress response and the intestinal microbiota composition of female forest musk deer (*Moschus berezovskii*) and their fawns. PLoS ONE.

[37] Wang Y., Smith J.A., Wilmers C.C. (2017). Residential development alters behavior, movement, and energetics in an apex predator, the puma. PLoS ONE.

[38] Schaller G.B., Hu J., Pan W., Zhu J. (1985). The Giant Pandas of Wolong.

[39] Zhang Z., Hu J., Han Z., Wei F. (2011). Activity patterns of wild red pandas in Fengtongzhai Nature Reserve, China. Ital. J. Zool..

[40] Yang N., Du Y., Liu M., Jiang Y., He X. (2025). The Spatiotemporal Distribution Patterns and Coexistence Mechanisms of Two Musk Deer Species in Mt. Gongga, Sichuan Province, China. Animals.

[41] Pradhan S., Saha G.K., Khan J.A. (2001). Ecology of the red panda Ailurus fulgens in the Singhalila National Park, Darjeeling, India. Biol. Conserv..

[42] Zhang Z., Sheppard J.K., Swaisgood R.R., Wang G., Nie Y., Wei W., Zhao N., Wei F. (2014). Ecological scale and seasonal heterogeneity in the spatial behaviors of giant pandas. Integr. Zool..

[43] Zhang J., Hull V., Ouyang Z., Li R., Connor T., Yang H., Zhang Z., Silet B., Zhang H., Liu J. (2017). Divergent responses of sympatric species to livestock encroachment at fine spatiotemporal scales. Biol. Conserv..

[44] Liu J., Ouyang Z., Taylor W.W., Groop R., Tan Y., Zhang H. (1999). A framework for evaluating the effects of human factors on wildlife habitat: The case of giant pandas. Conserv. Biol..

[45] Chen L., Zhang L., Zhao Y., He M., Wu H., Wang J., Chen Z., Zhao Y., Shen F., Zhang X. (2025). Impact of DNA methylation on digestive and metabolic gene expression in red pandas (*Ailurus fulgens*) during the transition from milk to bamboo diet. BMC Genom..

[46] Bista D., Baxter G.S., Hudson N.J., Murray P.J. (2023). Seasonal resource selection of an arboreal habitat specialist in a human-dominated landscape: A case study using red panda. Curr. Zool..

[47] Pei C., Li Y., Luo Z., Hai L., Lan X., Hu D. (2025). Diets and niche separation of forest musk deer (*Moschus berezovskii*) and roe deer (*Capreolus pygargus*) in spring in the Lyuliang Mountains, China. Chin. J. Ecol..

