# Supplementary material for: Response Strategies of Giant Panda, Red Panda, and Forest Musk Deer to Human Disturbance in Sichuan Liziping National Nature Reserve
Source: Biology (Basel). 2026 Jan 21;15(2):194. doi: 10.3390/biology15020194 (PMC12837811; doi:10.3390/biology15020194)
Supplement: Supplementary file 1 [file biology-15-00194-s001.zip › biology-4077871-supplementary.pdf]

Supplementary materials:

Table S1. Statistical Analysis of Peak Characteristics and Overlap in Daily Activity Rhythms of Giant Panda and Human Disturbances.

Species/Disturbance Type	Peak Time Period	Peak TRAI (95% CI)	Peak Overlap	Overlap Coefficient	Kolmogorov–Smirnov Test Result	Significance[1]
Giant Panda	14:00-16:00	0.854 (0.285-0.854)	-	-	-	-
Walking	12:00-14:00	15.091 (8.257-10.820)	Absent	0.032	D = 0.420, p = 0.0089	**
Gathering	16:00-18:00	1.424 (0.569-1.424)	Absent	0.047	D = 0.216, p = 0.7646	ns
Cattle	16:00-18:00	5.410 (2.847-4.271)	Absent	0.948	D=0.192, p=0.597	ns
Yak	08:00-10:00	1.424 (0.569-1.424)	Absent	0.939	D=0.300, p=0.208	ns
Goat	14:00-16:00	3.132 (1.424-2.563)	Present	0.953	D=0.207, p=0.509	ns
Horse	16:00-18:00	1.139 (0.569-1.139)	Absent	0.936	D=0.158, p=0.894	ns
Domestic Dog	10:00-12:00	0.569 (0.285-0.569)	Absent	0.845	D=0.378, p=0.324	ns

[1] *p < 0.05, **p < 0.01, ns = No significance.

Table S2. Statistical Analysis of Peak Characteristics and Overlap in Daily Activity Rhythms of Red Panda and Human Disturbances.

Species/Disturbance Type	Peak Time Period	Peak TRAI (95% CI)	Peak Overlap	Overlap Coefficient	Kolmogorov–Smirnov Test Result	Significance[1]
Red Panda	8:00-10:00	2.563（1.139-2.278）	-	-	-	-
Walking	12:00-14:00	15.091（8.257-10.820）	Absent	0.031	D = 0.297, p = 0.0029	**
Gathering	16:00-18:00	1.424（0.569-1.424）	Absent	0.038	D = 0.276, p = 0.2618	ns
Cattle	16:00-18:00	5.41（2.563-4.271）	Absent	0.918	D = 0.176, p = 0.185	ns
Yak	8:00-10:00	1.424（0.569-1.424）	Present	0.924	D = 0.158, p = 0.545	ns
Goat	14:00-16:00	3.132（1.424-2.563）	Absent	0.884	D = 0.253, p = 0.016	*
Horse	16:00-18:00	1.139（0.569-1.139）	Absent	0.906	D = 0.173, p = 0.459	ns
Domestic Dog	10:00-12:00	0.569（0.285-0.569）	Absent	0.819	D = 0.230, p = 0.748	ns

[1] *p < 0.05, **p < 0.01, ns = No significance.

Table S3. Statistical Analysis of Peak Characteristics and Overlap in Daily Activity Rhythms of Forest Musk Deer and Human Disturbances.

Species/Disturbance Type	Peak Time Period	Peak TRAI (95% CI)	Peak Overlap	Overlap Coefficient	Kolmogorov–Smirnov Test Result	Significance[1]
Forest Musk Deer	16:00-18:00	1.139 (0.569 - 1.139)	-	-	-	-
Walking	12:00-14:00	15.091 (8.257 -10.820)	Absent	0.024	D = 0.602, p <0.001	**
Gathering	16:00-18:00	1.424 (0.569 - 1.424)	Present	0.041	D = 0.512, p =0.005	**
Cattle	16:00-18:00	5.410 (2.847 - 4.271)	Present	0.906	D = 0.359, p =0.003	**
Yak	08:00-10:00	1.424 (0.569 - 1.424)	Absent	0.889	D = 0.370, p =0.011	*
Goat	14:00-16:00	3.132 (1.424 - 2.563)	Absent	0.852	D = 0.490, p <0.001	**
Horse	16:00-18:00	1.139 (0.569 - 1.139)	Present	0.906	D = 0.275, p =0.114	ns
Domestic Dog	10:00-12:00	0.569 (0.285 - 0.569)	Absent	0.736	D = 0.481, p =0.070	ns

[1] *p < 0.05, **p < 0.01, ns = No significance.

Table S4. Statistical Analysis of Overlap Coefficients and Chi-Square Test Results in Annual Activity Rhythms of Giant Panda and Human Disturbances.

Species/Disturbance Type	Overlap Coefficient	Chi-Square Test P-value	Significance[1]
Giant Panda	-	-	-
Walking	0.256 (0.145-0.658)	0.0005	**
Gathering	0.294 (0.000-0.635)	0.0020	**
Cattle	0.502 (0.117-0.656)	0.0025	**
Yak	0.627 (0.091-0.646)	0.2009	ns
Goat	0.461 (0.137-0.674)	0.0025	**
Horse	0.537 (0.094-0.655)	0.0095	*
Domestic Dog	0.067 (NA)	0.0005	**

[1] *p < 0.05, **p < 0.01, ns = No significance.

Table S5. Statistical Analysis of Overlap Coefficients and Chi-Square Test Results in Annual Activity Rhythms of Red Panda and Human Disturbances.

Species/Disturbance Type	Overlap Coefficient	Chi-Square Test P-value	Significance[1]
Red Panda	-	-	-
Walking	0.449 (0.330-0.710)	0.0005	**
Gathering	0.521 (0.085-0.621)	0.1124	ns
Cattle	0.701 (0.266-0.682)	0.0800	ns
Yak	0.723 (0.191-0.656)	0.1449	ns
Goat	0.695 (0.265-0.687)	0.0095	**
Horse	0.601 (0.195-0.677)	0.0095	**
Domestic Dog	0.048 (NA)	0.0005	**

[1] *p < 0.05, **p < 0.01, ns = No significance.

Table S6. Statistical Analysis of Overlap Coefficients and Chi-Square Test Results in Annual Activity Rhythms of Forest Musk Deer and Human Disturbances.

Species/Disturbance Type	Overlap Coefficient	Chi-Square Test P-value	Significance[1]
Forest Musk Deer	-	-	-
Walking	0.421 (0.249-0.667)	0.0005	**
Gathering	0.495 (0.083-0.647)	0.0630	ns
Cattle	0.641 (0.185-0.644)	0.1569	ns
Yak	0.716 (0.139-0.638)	0.1484	ns
Goat	0.626 (0.197-0.666)	0.0445	*
Horse	0.558 (0.134-0.632)	0.0085	**
Domestic Dog	0.037 (NA)	0.0005	**

[1] *p < 0.05, **p < 0.01, ns = No significance.
